# The Effects of Maternally Administered Methadone, Buprenorphine and Naltrexone on Offspring: Review of Human and Animal Data

**DOI:** 10.2174/157015908784533842

**Published:** 2008-06

**Authors:** W.O Farid, S.A Dunlop, R.J Tait, G.K Hulse

**Affiliations:** 1 School of Animal Biology, The University of Western Australia, Nedlands, WA 6009, Australia; 2 Western Australian Institute for Medical Research, The University of Western Australia, Nedlands, WA 6009, Australia; 3 School of Psychiatry and Clinical Neurosciences, The University of Western Australia, Nedlands, WA 6009, Australia

**Keywords:** Methadone, buprenorphine, naltrexone, pregnancy, fetal, neonatal, offspring, development.

## Abstract

Most women using heroin are of reproductive age with major risks for their infants. We review clinical and experimental data on fetal, neonatal and postnatal complications associated with methadone, the current “gold standard”, and compare these with more recent, but limited, data on developmental effects of buprenorphine, and naltrexone. Methadone is a µ-opioid receptor agonist and is commonly recommended for treatment of opioid dependence during pregnancy. However, it has undesired outcomes including neonatal abstinence syndrome (NAS). Animal studies also indicate detrimental effects on growth, behaviour, neuroanatomy and biochemistry, and increased perinatal mortality. Buprenorphine is a partial µ-opioid receptor agonist and a κ-opioid receptor antagonist. Clinical observations suggest that buprenorphine during pregnancy is similar to methadone on developmental measures but is potentially superior in reducing the incidence and prognosis of NAS. However, small animal studies demonstrate that low doses of buprenorphine during pregnancy and lactation lead to changes in offspring behaviour, neuroanatomy and biochemistry. Naltrexone is a non-selective opioid receptor antagonist. Although data are limited, humans treated with oral or sustained-release implantable naltrexone suggest outcomes potentially superior to those with methadone or buprenorphine. However, animal studies using oral or injectable naltrexone have shown developmental changes following exposure during pregnancy and lactation, raising concerns about its use in humans. Animal studies using chronic exposure, equivalent to clinical depot formulations, are required to evaluate safety. While each treatment is likely to have maternal advantages and disadvantages, studies are urgently required to determine which is optimal for offspring in the short and long term.

## 1 INTRODUCTION

The global average prevalence of heroin abuse in 2002-4 was 0.3%. However, there is considerable regional variation with, for example, an estimated prevalence of 0.1% in the United States, 0.2% in Africa, 0.7% in Russia, 0.8% in Europe, and as high as 4.4% in Iran [367]. Females make up a large proportion of heroin users. For example, based on estimates from the 2001 National Drug Strategy Household Survey in Australia, there are over 36,000 females (0.18% of the population) who use opiates either daily or weekly [277]. Approximately 80-90% of all women using intravenous heroin are of reproductive age [276, 291] and, although heroin can cause menstrual irregularities such as amenorrhea, oligomenorrhea and suppression of ovulation [108], pregnancy is not uncommon [313]. Indeed, a recent study showed that ~1/3^rd^ of patients presenting at a high risk clinic were positive for opioid use at some stage during their pregnancy [295]. In many countries, including the United States and Australia, oral methadone is the currently preferred maintenance pharmacotherapy for the pregnant heroin user [136, 190, 196]. However, recently, there have been a number of documented cases in which opioid-dependent women have undergone maintenance with oral naltrexone or sublingual buprenorphine for a portion, and in some cases the entire duration, of their pregnancy [158,  228,  248]. Moreover, with the advent of long-lasting, sustained-release naltrexone depot preparations and their potential use in treating heroin and alcohol dependence in women, an increasing number of fetuses are likely to be inadvertently exposed to naltrexone [149].

Although research on the developmental effects of methadone, naltrexone or buprenorphine already exists, it is inconclusive as to which treatment compromises fetal and postnatal development the least. The primary aim of this review is to provide a comprehensive overview of the developmental outcomes from exposure to methadone, buprenorphine or naltrexone during pregnancy and lactation in humans. Factors in addition to direct pharmacological actions of these drugs are also identified. To provide further insight into the independent effects of these pharmacotherapies, we review studies using pregnant animal models with discussion on mechanisms involved with opioid modulation of development.

### 1.1 Role of the Opioid System in Development 

Increasing evidence highlights the importance of the opioid system in mediating developmental events. Opioid receptor expression is regulated by signals from growth factors, proto-oncogenes and neurotransmitters [316]. Endogenous opioids and receptors are also present in the developing mammalian fetus. For example, endorphins are found in fetal plasma, and the classical opioid receptors, namely mu (μ), delta (δ) and kappa (κ), have been identified in the brain and other organs [134, 239, 360, 422, 423, 429]. Furthermore, opioid receptor expression [11, 229] and endogenous opioid concentration [11] in the fetus and neonate differ from that in adults. For example, the zeta (ζ) receptor (opioid growth factor receptor or OGFr), which belongs to the nuclear receptor superfamily, is ubiquitous in the developing rat brain, but unlike the classical receptors, it is not found in adults [174, 405, 434]. In addition, exogenously administered opiates such as methadone, have been localised in the brain of young rats, binding to μ-opioid receptors [299]. Moreover, higher methadone binding occurs in the fetal brain (2-14 times greater) compared to maternal neurons [299].

In a variety of species including mouse, rat, guinea pig and human, opioid levels and opioid receptor concentration fluctuate during development [11, 316]: universal opioid receptor binding is high during early embryogenesis, decreases towards birth, then gradually increases to adult levels [12, 22, 316]. Specifically, μ- and κ-receptors appear in the embryonic brain, whereas (-receptors appear later [432] with a similar time course being seen in humans and rats [244]. Furthermore, the receptor increases are associated with increases in endogenous opioid levels [11].

Endogenous opioids serve as natural inhibitory trophic factors [405], inhibiting mitosis and DNA synthesis in the developing brain [293, 429]. Whilst the role of κ-receptors is not clearly understood, selective μ-receptor activation has been demonstrated to inhibit proliferation, whereas selective δ-receptor agonists inhibit neuronal differentiation [132]. Cell viability and death are also controlled by opioid-mediated signalling [359], of which opioids have a tendency to suppress viability and increase cell death, perhaps the underlying cause for smaller opioid-exposed newborns [152, 341].

The ζ-receptor has been shown to play a prominent role in the mediation of fetal cell proliferation [442], the biology of which has received thorough review [440]. Receptor function is regulated by its endogenous ligand, [Met^5^]-enkephalin (opioid growth factor or OGF), which has an inhibitory effect on transcription [433]. In human and rat, it is expressed during development in the cerebrum and cerebellum, and in somatic regions, for example heart, muscle and bone [440]. Developmental delays observed after fetal exposure to agonists, such as heroin or methadone, are postulated to result from their direct interaction with OGFr, retarding “normal” fetal development. On the other hand, fetal exposure to an opioid antagonist, (e.g. naltrexone) may prevent [Met^5^]-enkephalin from binding to the opioid growth factor receptor, thereby blocking the growth-inhibitory effects of the OGF-OGFr complex [405, 435, 440, 441]; in other words, the brake is removed and growth is accelerated. Other studies have confirmed that OGFr localisation is associated with proliferating cells in the brain [174, 404, 442]. Furthermore, this receptor also up-regulates in response to decreased agonist release and receptor binding during cell proliferation and growth [431].

## 2 TREATMENT FOR THE PREGNANT HEROIN USER

Heroin (diacetylmorphine) is an analogue of morphine which efficiently crosses the blood-brain barrier (BBB) [27] and its active metabolic products elicit morphine-like effects *via* agonist activity at the μ-opioid receptor [309]. The abuse liability of intravenous heroin is attributable to its potency, rapid crossing of the BBB and induction of effects. The elimination half-life of heroin and its active metabolites is short (15-30 mins) [27]; tolerant users are therefore likely to use heroin several times each day to prevent aversive withdrawal symptoms [168, 213]. Whilst pregnancy can be a powerful motivator for ceasing drug abuse, many pregnant women revert back to dependent heroin use, albeit commonly at a reduced level, and relapse post birth is common [104, 127, 203, 272, 295]. Heroin readily crosses the placenta [232] and, consequently, maternal heroin use during pregnancy is associated with an increased risk for a number of adverse neonatal outcomes, including low birthweight, antepartum haemorrhage and increased neonatal mortality [194, 222, 237, 373, 391], many of which have been confirmed in animal studies [109, 223, 401, 419, 444]. Meta-analyses on neonatal outcomes and maternal heroin use have produced crude odds ratios for antepartum haemorrhage (2.33; 95% CI 1.32-4.30) [153], low birthweight (4.61; 95% CI 2.78-7.65) [152] and neonatal mortality (3.27; 95% CI 0.95-9.60) [154]. In addition, compared to non-users, women who continue to use heroin during pregnancy have a significantly increased risk of exposure to infection and negative lifestyle factors associated with acquiring and injecting heroin, as well as a significantly poorer attendance at antenatal or other health care services [54, 222, 237]. 

### 2.1 Methadone

#### 2.1.1 Pharmacology and Role of Methadone in Treatment of Dependence

Methadone (6-dimethylamino-4,4-diphenyl-3-heptone) is a synthetically derived agonist which selectively binds to the μ-opioid receptor, thereby exerting morphine-like effects. Currently, methadone maintenance treatment (MMT) is the most commonly used pharmacotherapy for opioid-dependence [325]. Methadone has a high bioavailability of 40-99% [101, 214, 260, 284, 285, 325, 330], and a relatively long elimination half-life of approximately 24 hours [164, 168, 171, 397]. It therefore prevents opioid-withdrawal syndrome in opioid-dependent patients when they are maintained on a single daily oral dose of 60-100 mg [56, 79, 84, 86, 168, 384] MMT falls within the realm of harm minimisation, and its use results in reductions in illicit drug use, crime, morbidity and mortality [213, 370, 385, 386, 388]. However, as methadone is an agonist, use of other opiates can still elicit morphine-like effects, with fatal overdose as a potential outcome [24, 247, 368]. For example, although sufficient methadone reduces or eliminates drug craving [213, 384, 393], in patients who fail to remain abstinent, methadone may attenuate the effects of, for example, heroin through cross-tolerance and increased competition for binding at μ-opioid receptor sites [76, 107, 311, 384]. Another limitation with this pharmacotherapy is that the duration of withdrawal is protracted compared to withdrawal from heroin; consequently, detoxification from methadone has lower success rates compared to detoxification from other opioids [4, 69, 75, 117, 118, 311].

#### 2.1.2 Role of Methadone in Treatment of Dependence During Pregnancy

In many countries, including Britain, the United States and Australia, MMT is the currently accepted management for the pregnant heroin user. Patient compliance to MMT during pregnancy is generally high compared to non-pharmacotherapeutic interventions. Furthermore, higher methadone doses are associated with improved compliance, particularly during the last trimester of pregnancy [67, 95, 187, 220, 356]. As MMT involves daily dispensing only by licensed personnel, continual contact with the patient is accomplished and is associated with improved antenatal care [221]. In addition, toxicological screening for illicit drugs in pregnant patients undergoing MMT indicate that increasing methadone doses are associated with decreased drug abuse [23, 196, 251]. Thus, MMT upholds the policy of harm minimisation by reducing heroin use and other associated negative life-style factors, both of which improve neonatal outcomes. However, caution is required in selecting the maintenance dose for managing opioid-dependent pregnant women as methadone crosses the human placenta [329] and is currently registered by the Food and Drug Administration (FDA) in the United States as a Category C drug, a classification which is similar to that adopted by the Therapeutic Goods Administration (TGA) in Australia (also Category C) (Table **1**). 

#### 2.1.3 Pharmacokinetics of Methadone During Pregnancy

In addition to the benefits derived from MMT, methadone exposure also directly affects the fetus and newborn, the impact of which is dictated by pharmacokinetics during pregnancy and the extent of transfer to offspring. Metabolic inactivation of methadone to its primary metabolite, 2-ethyl-idene-1,5-dimethyl-3,3-diphenylpyrrolidine (EDDP) involves N-demethylation, mainly by hepatic cytochrome P_450_3A4 (CYP3A4) [28, 269, 325]. To a lesser extent, the microsomal enzymes, CYP-2D6, -1A2, -2B6, -2C9, and -2C19 [86, 100, 111], also contribute to methadone inactivation. Furthermore, human intestinal microsomes have an additional metabolic role, transforming methadone sequentially to EDDP and 2-ethyl-5-methy-3,3-diphenyl-pyrroline (EMDP) [289]. As pregnancy progresses, methadone metabolism becomes enhanced, manifesting as increased clearance [178, 396], with a reduced elimination half-life of 8-20 hours compared to the 24 hour half-life in a non-pregnant patient [105, 306, 357]. Thus, as pregnancy progresses, lower methadone plasma concentrations result despite an unchanged dose [178, 212, 306, 357, 396]. The reduction in methadone blood levels as pregnancy progresses may also be attributable to the slight increase in the volume of distribution, lowered plasma protein binding and decreased oral absorption [105, 178, 306]. 

Methadone blood levels late in pregnancy also require consideration. Dosing is highly variable, being 15-100 mg per day [43, 61, 74, 85, 112, 115, 192, 198, 246, 249, 251, 324, 339, 398]. Methadone at 240 ng/ml or greater in the maternal circulation was recently identified as the required level for preventing maternal withdrawal during the third trimester, and is achieved by an oral dose of 50-150 mg [78]. Lower levels are associated with increased reporting of withdrawal symptoms which, if not addressed by the clinician, can lead to patient relapse to illicit opiate use, which in turn is detrimental to the well-being of the individual and their developing offspring [78, 178, 306]. 

Drug transfer from the maternal to the fetal circulation is accomplished *via* the placenta which also acts as a barrier. Placental permeability is determined by the number of tissue layers between the fetal and maternal blood, the relative relationship of fetal to maternal blood flow, and the recruitment of specialised transport processes, such as facilitated and active diffusion. Methadone is also retained by the placenta and does not have adverse effects on its viability and function [281]. Furthermore, the placenta can metabolically alter many drugs during transfer. Methadone crosses the human placenta [329] and is dependent upon three processes: metabolism, simple diffusion, and carrier-mediated uptake/efflux [274]. 

Metabolic inactivation of methadone to EDDP is mediated by the placental enzyme, aromatase (CYP19) [138], but EMDP has not been detected in trophoblast tissue, indicating that sequential demethylation does not occur [275]. Placental metabolism allows tonic regulation of methadone transfer to the fetus, although inactivation by the placenta is significantly less than that of the maternal liver or intestine [28, 100, 275, 289]. During gestation, the placenta also undergoes continual structural and functional changes, which may lead to modulated activity and expression of metabolic enzymes. Whilst affinity of placental aromatase for methadone is unchanged between 17- and 34-weeks gestation (2^nd^ to mid-3^rd^ trimester), aromatase concentrations increase 4-fold and catalytic activity doubles at term (37 weeks) [138, 275], in part accounting for lower methadone blood levels as pregnancy progresses. Another factor contributing to the difficulty in selecting appropriate doses is the 4- to 6-fold individual variation in the rate of methadone metabolism by the placenta at term [275].

Methadone transfer is also bi-directional due to dual perfusion of the placental lobule. The transfer and clearance index for methadone is greater in the returning fetal to maternal circuit compared to the maternal to fetal direction due to unidirectional activity of the efflux transporter, P-glyco-protein, localised in the brush-border of trophoblast cells [274, 281].

There have been a limited number of studies which have compared methadone levels in the fetus and neonate and its association with the period of pregnancy, maternal levels, and maternal dose [105]. The studies support extensive materno-fetal transfer of methadone with considerable levels in cord blood [281, 324], although these are approximately 5-fold less than the maternal plasma concentration [128]. Once in the fetus, methadone undergoes hepatic metabolism, the capacity of which depends on fetal maturation, with CYP-3A4 and -2D6 being present by week 20 [125, 219]. However, fetal metabolic capacity is considerably less than in the mother, due to the absence of other cytochrome enzymes, including CYP-2C9, -1A2 and -2B6 [125, 310]. As pregnancy progresses, fetal metabolic capacity increases but at birth decreases dramatically [250, 314, 396]. In addition, methadone has been identified in amniotic fluid, suggesting oral and cloacal uptake, although such exposure is presumably minimal compared to that received across the placenta [128, 211, 212, 326]. 

Methadone is also transferred into breast milk, thereby exposing infants postnatally. The pharmacokinetic parameters involved with methadone transfer into breast milk have been reviewed elsewhere [176, 252]. Milk-to-plasma ratios vary considerably between studies, ranging from 0.05 to 1.89 [25, 212]. With an average breast milk intake of 475 ml/day and a large maternal dose range (10-180 mg/day), the estimated neonatal/infant intake of methadone *via* the breast milk ranges widely (0.01-0.1 mg/day). [25, 110, 212, 252]. The elimination half-life in neonates is longer (32.5 h) than in adults (24 h) [212], contributing to higher levels on a mg per kg basis [105, 281, 324]. Nevertheless, the relative infant dose has been estimated as 2.8% of the maternal one [16]. Despite the relatively low level of infant exposure to methadone, caution is required for lactating women undergoing MMT as animal studies have demonstrated altered postnatal development [256, 257, 409-411, 418, 420]. 

#### 2.1.4 Methadone and Developmental Effects

##### 2.1.4.1 Methadone and Fetal Outcomes

Methadone has undoubtedly improved the management of pregnancy and neonatal outcome in heroin addicts [196]. For example, high methadone doses in the third trimester are related to improved fetal growth compared to pregnancies of non-treated heroin addicts [124]. Importantly, no serious fetal toxicity has been associated with methadone [81, 222]. However, human studies have identified adverse effects associated with methadone use during pregnancy, although many of these findings are inconsistent and have not established a causal link to methadone use. Poor outcomes include a higher incidence of prematurity [6, 85, 324], intrauterine growth retardation [6, 74, 195, 198, 324] and increased fetal mortality [198, 242]. Clinical observations *in utero* have also shown that fetal activity, respiration and heart rate are depressed in mothers taking daily oral methadone compared to those not dependent on opioids [175, 307, 398].

##### 2.1.4.2 Methadone and Neonatal Outcomes

Methadone maintenance treatmentin the stabilised patient, when combined with antenatal care and supervision of illicit drugs, produces a considerable improvement in birthweight and decreases the risk of neonatal morbidity, when compared to the outcomes of neonates from non-treated pregnant heroin users [88, 196, 198, 350, 373]. However, MMT during pregnancy is still associated with poorer neonatal outcome compared to drug-free controls. For example, the proportion of infants of low birthweight ranges from 29-62% (mean: 45%; n=730) for heroin [88, 102, 312, 350, 351, 443], 18-47% (mean: 29%; n=845) for methadone [88, 129, 180, 283, 350, 443], and 3-15% (mean: 15%; n=42019) for drug-free controls [88, 102, 129, 180, 351]; these studies, however, did not consistently control for confounding factors, such as other drug use. Additionally, when compared to drug-free controls, MMT during pregnancy is associated with reduced head circumference [33, 74, 231] as well as slight increases in neonatal mortality [54, 154, 194], morbidity and the incidence of neonatal abstinence syndrome [6, 61, 80, 129, 191, 324]. 

In comparison to low dose MMT, birthweight and head circumference is improved by higher methadone dosing during the early and late phases of pregnancy [74, 124, 195]. Such improvements in neonates are presumed to be a consequence of better lifestyle factors (e.g. nutritional intake) and reduced drug abuse. Indeed, human studies using lower methadone doses, which were insufficient to alleviate withdrawal symptoms, were associated with increased abuse of other drugs [23, 196, 251]. Furthermore, a recent investigation on human development following *in utero* exposure to methadone demonstrated dose-related growth effects contrary to that previously reported, whereby, neonates from patients administered methadone during pregnancy for the management of opioid-dependence were compared with newborns of patients who received methadone for the management of pain [339]. The dosing regimens differed in that patients being managed for pain received lower doses of methadone (40 mg/day) and for a shorter duration of time (5 weeks toward end of pregnancy) in comparison to heroin users undergoing MMT, who received 60 mg per day for a duration of 36 weeks [339]. As pain-managed patients were not opioid-dependent prior to receiving methadone, they were less likely to use other drugs of abuse than the cohort of opioid-dependent patients, when methadone reached trough levels. This effect is in part due to their lower levels of tolerance from an abstinent history of opioid use. Thus, it may be proposed that these data reflect the true pharmacological actions of methadone, whereby higher doses are associated with a greater suppressant effect on growth.

###### 2.1.4.2.1 Methadone and Neonatal Abstinence Syndrome

Neonatal abstinence syndrome (NAS) is characterised by high-pitched crying, hyperactive reflexes, tremors, hypertonicity, convulsions, frantic suckling of fists, poor feeding, regurgitation, diarrhoea, dehydration, yawning, sneezing, nasal stuffiness, sweating, skin mottling, fever, rapid respiration and skin abrasion, as assessed by the Finnegan scale [91, 128, 191, 194, 324]. Although symptoms can lead to prolonged hospital stays for the new born [183], behavioural abnormalities do not persist. However, withdrawal following MMT is likely to be more prolonged than that from heroin [177] and, if NAS is left untreated, there is an increased risk of mortality [88, 304]. Consequently, some physicians are reluctant to increase the daily methadone dose as pregnancy progresses, despite the increased clearance and volume of distribution and, therefore, reduced plasma concentrations and declining patient satiety. Moreover, some highly motivated patients are encouraged to endure gradual dose reductions to accomplish complete detoxification prior to parturition [60, 215, 246, 294]. However, this approach is generally avoided as the post-detoxification risk of relapse [92] is likely to involve fluctuating opiate levels, leading to numerous complications, such as fetal distress, meconium aspiration, fetal hypoxia and death [90, 177, 190, 447]. As NAS can be managed pharmacologically, stabilisation onto methadone, involving sufficient dosing and employment for the duration of gestation, is the preferred management for the pregnant opioid-dependent woman [190, 191, 294].

The incidence of NAS when pregnant opioid-dependent women receive MMT is typically 60-90% of newborns [33, 129, 189, 194, 228, 324]; a large proportion of which (45-80%) endure severe withdrawal, necessitating pharmacotherapeutic intervention to alleviate the symptoms [13, 23, 80, 195, 246, 339]. The issue of maternal methadone dose versus the prognosis of NAS remain unresolved; whilst a number of studies show a positive correlation between dose and the prevalence, duration and severity of NAS [61, 73, 246, 294, 339], others report the absence of this association [23, 33, 194, 243, 283, 324]. The inconsistency is presumably from the inability to appropriately control for numerous confounding factors, such as poor antenatal care, smoking, poverty, personal neglect, and poor health status [364]. For example, a recent study demonstrated that the onset and severity of NAS was greater in neonates from patients smoking more than 20 cigarettes per day versus less than 10 per day [46]. Other studies do not sufficiently control for cigarette smoking [1, 129, 246, 249], or other drugs which can influence the prognosis of NAS [112, 191, 339]. Another study showed that higher methadone doses in third trimester were associated with increased gestational age, and after adjustments were made for maternal dose and gestational age, a correlation was found between NAS, gestational age and race, with non-black infants exhibiting higher NAS scores than black infants [124]. 

The rate of decline in neonatal methadone plasma concentration is positively correlated with the severity of NAS during the first four days after birth [73]. Another study which examined neonatal plasma concentration and NAS reported that neonatal withdrawal was not observed if their methadone plasma concentration was greater than 60 ng/ml [324]. This finding is consistent with recent data whereby maternal withdrawal is avoided when methadone plasma levels are greater than 240 ng/ml [78]. Indeed, it has been shown that maternal and fetal withdrawal occur simultaneously [2], even though perinatal methadone blood levels are approximately 5-fold less than maternal ones [128]. The findings provide a plausible explanation for observed decreases in the severity of NAS after exposing the newborn to methadone *via* the breast milk [1, 9, 245, 252]. This approach may therefore be useful in the management of the newborn displaying symptoms of withdrawal.

Improved management of the pregnant opioid-dependent person may be achieved by MMT with split dosing, whereby half the standard methadone dose is administered twice-daily [196, 252, 357]. Compared to single-daily dosing, split-dosing more closely resembles a steady state plasma concentrations over a 24 hour period [395]. Furthermore, methadone is sustained at a higher concentration and for longer from a split rather than a once-daily dose [110, 196, 357]. In addition, compared to single-dosing, split-dosing during the third trimester of pregnancy is associated with reduced cocaine abuse and improved compliance [67]. It is therefore suggested that split-dosing will improve compliance and treatment by producing fewer withdrawal symptoms [357], reducing depression of fetal body movements and breathing rate [175]. However, there have only been a few investigations into the benefits of split-dosing during pregnancy; although, based on these preliminary findings, further studies are warranted.

##### 2.1.4.3 Methadone and Postnatal Outcomes

Postnatal effects from MMT exposure during pregnancy include reduced postnatal weight gain, head circumference and height [42, 127, 346, 353, 365]. However, although opiate-induced growth reductions have a tendency to diminish with age [226, 231, 446], they can persist for up to 5.5 years [369]. A weak association has also been made between MMT and sudden infant death syndrome, although a causal effect is yet to be established [89, 142, 197, 304]. Other postnatal effects include an increase in the incidence of mortality [33, 221, 346], microcephaly [6], strabismus [112] and behavioural effects such as mood, attention and cognitive deficits [13, 43, 115]; behavioural outcomes following prenatal exposure to opiates have previously been reviewed [193]. It was recently shown that at four months of age, a highly significant difference in neurological development exists between control infants, and infants exposed to methadone during the prenatal period, with methadone-exposed infants displaying a 14% greater latency in reaching peak visual evoked potentials (VEPs), in response to changing patterned visual cues [392]. Infants of mothers who underwent MMT during pregnancy display higher scores for disorganised and avoidant behaviour, as well as lower scores of contact-maintaining behaviour at 12 months of age [115, 127]. Furthermore, infants of drug-dependent mothers receiving methadone had delayed cognitive development, persisting in up to 50% of these infants until 5 years of age [369]. These infants are also less interactive at 5 years of age compared to the normal population. In addition, 7 year old methadone-exposed infants differed from controls in their behaviour at school, displaying poorer achievement, increased aggression and school disruptions [323]. However, other reports found no difference between methadone-exposed, heroin-exposed and control infants aged 3 to 5 years with respect to cognitive and social function [115, 127, 192, 231, 352]. 

#### 2.1.5 Concomitant Heroin Use During Methadone Maintenance Treatment

Although heroin-using women who stabilise on methadone at or around the time of conception show good prognosis, those who fail to stabilise and continue regular heroin use throughout pregnancy show significantly poorer prognosis [152-154]. For example, a meta-analysis of neonatal birthweight showed a lower relative risk estimate for women stabilised on methadone at or near the time of conception (RR 1.36; 95% CI 0.83-2.22) compared to those who failed to stabilise and continued to use heroin (RR 3.28; 95% CI 2.47-4.39) [154]. Despite improvements over those continuing to use heroin, neonatal outcomes such as birthweight and delivery at term for those on MMT are significantly poorer than for matched controls who do not use heroin [152, 155]. Furthermore, neonates/infants of those treated with methadone who also co-use illicit opiates are at increased risk of death [154]. Similarly, there is an increased likelihood of infants developing NAS when mothers undergoing MMT supplement with heroin [61]. 

There are major improvements associated with MMT in the risk of exposure to infection, negative lifestyle factors, reduction of heroin use, antenatal attendance and neonatal outcomes such as antepartum haemorrhage, low birthweight and neonatal mortality [139, 152-154, 194, 221]. However, there are adverse effects associated with MMT during pregnancy which may lead some clinicians to consider alternative treatments. The critical issue in improving outcomes for opioid-dependent pregnant women is either achieving a stable intra-uterine milieu, with minimal intoxication and withdrawal, or minimising exposure to opioids and the associated potential growth-suppressive effects. Clinical evidence suggests greater benefit from the former. Whilst abstinence from any drug is ideal during pregnancy, detoxification from MMT might not be ideal if the consequences include a reversion to illicit opiate or other drug use. Although detoxification from methadone before parturition can be accomplished in highly motivated patients [60, 155], continued illicit drug use is likely and the high level of maternal complications suggests that improvements are needed in the treatment of pregnant heroin users [198].

### 2.2 Buprenorphine

#### 2.2.1 Pharmacology and Role of Buprenorphine in Treatment of Dependence

Buprenorphine ([9-cyclopropylmethyl-4,5-epoxy-6,14-ethanomorphinan-7-yl]-3-hydroxy-6-methoxy-3,3-dimethyl-butan-2-ol) is a semi-synthetic thebaine derivative which selectively binds as a partial agonist to the μ-opioid receptor, and to the κ-opioid receptor as an antagonist [225, 230, 280], although the latter role is unclear as a more recent study implicates agonistic activity at the κ-opioid receptor in relation to analgesia [303]. Buprenorphine was first evaluated for its potential in maintaining the opioid-dependent person in 1978 [179], although it was not approved by the United States legislature until 2000, and by the FDA until 2002 [98]. As a partial agonist, buprenorphine potently induces morphine-like effects at a relatively low-dose, but at higher doses, it has a submaximal response compared to full agonists, such as morphine and heroin, and begins to attenuate in its agonistic effect [230, 308]. Buprenorphine also attenuates the effects of other opioid agonists, through increased competition for binding at the μ-opioid receptor [355]. Sublingual buprenorphine is rapidly absorbed and distributed, with a bioavailability of approximately 50% (range: 16-94%), which is marginally less than the bioavailability of methadone [36, 52]. However, it slowly dissociates from the opioid receptor binding sites resulting in a biphasic elimination half-life of approximately 30 hours with an initial rapid phase of 3 to 5 hours, followed by a slow phase greater than 24 hours [35, 36, 52, 216]. 

Buprenorphine is a highly lipophilic agent which is extensively metabolised in the liver by N-dealkylation to norbuprenorphine; similar to methadone, the process is mainly (~75%) mediated through CYP3A4 [53, 172, 205], with the remaining ~25% mediated by CYP-3A5, -3A7 and -2C8 [172, 205, 270]. The active metabolite, norbuprenorphine, along with its parent compound, undergo inactivation by conjugation with glucuronic acid [53, 290].

The regimen commonly employed clinically is daily sublingual administration (Subutex®) [30, 363]; buprenorphine’s efficacy has been reviewed elsewhere [62, 207, 235, 236]. Buprenorphine doses used to manage the opioid-dependent person vary considerably (mean dose range: 1-24 mg/day) [184, 208, 234, 355, 363]. In comparison to lower doses (1-4 mg/day), the typical dose range of 8-16 mg/day is associated with improvements in patient compliance and abstinence from other opioids [236, 332, 333, 355]. However, its effect in reducing abuse of non-opioid drugs, such as cocaine, is less pronounced [119, 333, 389]. Further benefit of buprenorphine for managing addiction resides in its partial agonist properties since reduced liability for abuse, accidental overdose and adverse effects are attributed to buprenorphine’s ceiling effect, whereby higher doses are associated with much smaller increases in morphine-like effects [10, 30, 230, 236]. In addition, withdrawal is generally well tolerated, being less severe than for heroin or methadone [114, 238, 355]. Despite its advantages, buprenorphine carries the potential for abuse [30]. However, the Suboxone® formulation prevents abuse since buprenorphine is combined with the short acting μ-opioid receptor antagonist, naloxone [3]. When taken sublingually, the poor bioavailability of nalo-xone (<10%) results in minimal interference with systemic buprenorphine; however, if taken intravenously, naloxone blocks the pleasurable effects of buprenorphine [97, 130, 259]. 

#### 2.2.2 Role of Buprenorphine in Treatment of Dependence During Pregnancy

The benefits of buprenorphine maintenance of the nonpregnant heroin user are also seen in pregnancy [286], with retention to treatment, reduced illicit drug use, and improved safety with regards to overdose [185, 188, 189]. The TGA and FDA have classified buprenorphine into the same pregnancy category (C) as methadone [38] (Table **1**), although, its use as a maintenance treatment during pregnancy is restricted due to a lack of data. Nevertheless, human studies have been conducted where FDA restrictions do not apply, including France, Belgium and Austria. In France, buprenorphine has been used as a standard treatment for opioid-dependence for over a decade. Whilst some women continued buprenorphine maintenance after conceiving, others underwent detoxification from heroin, followed by induction onto buprenorphine [5, 228, 286]. Buprenorphine doses used to maintain the pregnant woman are variable, with a mean dose range of 5.3-18.7 mg/day [93-95, 186, 189, 218, 228, 331]. Similar to methadone, buprenorphine is transferred across the placenta to the neonate [273], although this occurs to a lesser extent, presumably due to buprenorphine’s greater molecular weight (504.1 Da).

#### 2.2.3 Pharmacokinetics of Buprenorphine During Pregnancy

Also in parallel with methadone, buprenorphine is metabolised by the placenta [70, 273]. Transfer of buprenorphine and norbuprenorphine across the placenta is evidenced by its detection in umbilical cord and neonatal blood, urine and meconium, with cord levels ranging 101-137 ng/ml [186, 248]. The placenta can also accumulate buprenorphine, thereby acting as a depot and allowing slow dissociation to the fetus [273]. Nonetheless, placental metabolism, mediated *via* the placental enzyme aromatase (CYP19), reduces the amount of transferred [70, 273]. A further reduction in fetal exposure is mediated by the efflux transporter molecule P-glycoprotein [282]. There are low levels of CYP3A4 in fetal liver and so fetal metabolism does occur, although this is minimal. However, buprenorphine metabolism is also mediated through CYP3A7 [125, 400] which would account for the higher neonatal plasma levels at term compared to maternal trough levels [248]. During lactation, buprenorphine is transferred *via* breast milk at a similar concentration to that found in maternal plasma (0.230 to 0.720 ng/ml) but this level of neonatal exposure is considered to be negligible [186, 248]. 

#### 2.2.4 Buprenorphine and Developmental Effects

##### 2.2.4.1 Buprenorphine and Offspring Outcomes

Clinical retrospective and prospective studies on buprenorphine maintenance for pregnant opioid-dependent women indicate that it is a well tolerated and safe [93, 185, 188, 228]. Neonatal outcomes are not conclusive as studies are limited [93, 186, 228]. Nevertheless, most pregnancies lack complications with neonatal outcomes, including birthweight, APGAR scores, head circumference and body length, being within normal ranges [93, 94, 331]. In addition, mortality is not a major problem, with one stillbirth and one spontaneous abortion being the only reported cases [218]. Notwithstanding the benefits of managing pregnant opioid-dependent women with buprenorphine, caution is needed as adverse effects have been associated with this treatment. For example, one study reported that 10% of buprenorphine-exposed newborns (of 259) were delivered prematurely, compared to a normal incidence of 7% [228]. Furthermore, another study reported that 5.8% (2/34) newborns had malformations [218]. Follow-up postnatal studies are also scarce, although in a recent study, neurological development of infants exposed to buprenorphine during gestation was shown not to differ from control infants at four months, as assessed by measurement of VEP latencies [392]. It is of concern that lower limb hypertonia, jerky movements and jitteriness in some newborns have been reported to last for 3-9 months [201], although these may be a consequence of maternal poly drug use during pregnancy and/or lactation [126, 377, 378].

##### 2.2.4.2 Buprenorphine and Neonatal Abstinence Syndrome

Neonatal abstinence syndrome is apparent in only a few buprenorphine-exposed newborns, a feature considered to be a major advantage of this drug treatment during pregnancy, [94, 185, 218]. However, one study of 13 neonates reported a NAS incidence rate of 85% [201]. Nonetheless, in most cases, NAS presented as mild and did not require treatment [94, 185, 218]. This may, at least in part, be explained by the extensive metabolism of buprenorphine within, and relatively low transfer across, the placenta. Symptoms appear at 12 hours, peak at around 72 hours and alleviate at 120 hours after the last buprenorphine dose [95, 186], although this timing could be confounded by the use of other drugs, such as tobacco [46]. Plasma levels above 0.7 ng/ml reportedly prevent withdrawal [217]. There are currently no evidence-based guidelines for breastfeeding in women maintained on buprenorphine during pregnancy and breast feeding due to lack of conclusive data [94, 185, 228, 248]. However, breastfeeding occurs and buprenorphine, regardless of maternal dose, does not alter the effectiveness of using morphine to treating NAS [116, 186]. 

#### 2.2.5 Sublingual Buprenorphine Versus Methadone Maintenance

A number of investigations have made comparisons between neonatal outcomes from buprenorphine or methadone maintenance during pregnancy. A buprenorphine dose of 8 mg/kg is comparable to a methadone dose of 60 mg/day with respect to efficacy in treating addiction and reducing drug abuse [236]. MMT is associated with lowered use of additional opioids, as indicated by random toxicological screening, whereas buprenorphine is associated with greater patient compliance [95, 389]. Whilst buprenorphine is less toxic to the fetus than methadone [288], the increased use of other drugs during the buprenorphine-maintained pregnancy may produce worse outcomes for the neonate [82, 126, 151]. 

In general, however, management with buprenorphine during pregnancy is comparable to MMT, with regards to safety of the neonate. In 2006, a study with 259 participants did not identify any major differences for perinatal outcome between methadone (57+/-30.4 mg) and buprenorphine (5.4 +/- 4.5 mg) [228]. However, the peak NAS severity was observed earlier in newborns exposed to buprenorphine during gestation, compared to newborns exposed to methadone (66 *versus* 81 h) [228]. Conversely, the onset of NAS was earlier following MMT (60 h) compared with buprenorphine (72 h) [95], which may be explained by the longer terminal half-life of buprenorphine [35, 36, 52, 216]. Furthermore, NAS is generally more severe following MMT [228], as indicated by a higher amount of morphine required to treat NAS in comparison to the amount required for treating buprenorphine-exposed infants [189]. Reduced NAS in buprenorphine exposed infants may be due to decreased placental transport. As discussed earlier, VEP latencies of infants exposed to methadone were significantly longer than control infants, whereas maternal use of buprenorphine during pregnancy resulted in infant VEPs similar to controls, conferring an advantage over methadone [392].

### 2.3 Naltrexone

#### 2.3.1 Pharmacology and Role of Naltrexone in Treatment of Dependence

Naltrexone (N-cyclopropylmethyl-noroxymorphone) is a thebaine-derivative that binds non-selectively to opioid receptors as an antagonist, its pharmacological action in blocking the effects of opioids (such as methadone, morphine and heroin) may be useful to prevent relapse in opioid-dependent patients [113, 120]. Systemic naltrexone efficiently crosses the human blood-brain barrier [96, 199, 227, 267]. Compared to other antagonists (such as nalorphine and naloxone), naltrexone has a long duration of action (elimination half-life: ~4 hours) and greater antagonist potency [380, 402]. Naltrexone is principally eliminated by the liver, where metabolic (NADPH-dependent) reduction of its 6-keto group produces 6,β-naltrexol [26, 266, 381]. Additional hepatic metabolism is mediated by glucuronic acid conjugation and catechol-O-methyl transferases, resulting in the formation of a number of minor metabolites which are excreted primarily by the kidney [40, 266, 375, 380-382]. In humans, plasma naltrexone concentrations are 10 times less than that of its primary metabolite, 6,β-naltrexol [40] which has about a 1/100 the antagonistic potency of naltrexone at the μ-opioid receptor [39, 170]. The ability of naltrexone to effectively antagonise heroin use has been clearly established, but the exact level required is still in question. It has been shown that serum naltrexone blood levels of 2.8 ng/ml block 500 mg of snorted pure pharmaceutical diacetylmorphine [32], ≤2ng/ml blocks the effects of 25 mg of intravenously administered heroin [279, 376] and ≤1 ng/ml antagonises 15 mg morphine [45]. The generally cited therapeutic blood level for the management of heroin dependence is 2 ng/ml [32, 50, 156, 264], which is 87% effective in blocking the effects of 25 mg intravenously administered heroin [51], although higher values may be needed to provide 100% coverage. Notwithstanding the poor oral bioavailability (40%) of naltrexone, it has been available for many years as an oral formulation (ReVia®), commonly involving daily self-administration (50 mg) to maintain therapeutic blood levels, although other prescribing patterns, such as 100 mg three times per week are also used [264]. The standard oral dose of one 50 mg tablet per day produces a peak of ~8 ng/ml of blood naltrexone, decaying to 1.1 ng/ml within 24 hours [380]. However, oral naltrexone is associated with poor compliance [21, 328].

An alternative to oral naltrexone involves either injection of a depot formulation or surgical insertion of a sustained-release implant. Either approach removes the onus on patients to use oral naltrexone on a daily basis. Several sustained-release naltrexone implants have been developed which maintain therapeutic blood levels (≥2 ng/ml) for 4-8 weeks [29, 32, 49, 209, 263, 362]. Depotrex® (192 or 384 mg naltrexone) is an injectable depot formulation which antagonises the effects of intravenously-administered heroin (0-25 mg) for 3-5 weeks, and is safe and well tolerated in opioid abusers, supporting its use as a treatment for opioid dependence [50]. Similarly, Vivitrol® (formally known as Vivitrex) is an injectable depot formulation which produces peak naltrexone plasma levels within 3 days, dropping to an undetectable level (≤ ng/ml) by 35 days [15]. Preliminary data indicate that in humans, intramuscular Vivitrol is also well tolerated with reported adverse effects being mild to moderate [106]. 

However, although sustained-release injectable and implantable preparations help overcome the issue of daily medication and non-compliance, they only last for short periods and still incur significant levels of non-compliance. For example, in an 8-week Depotrex trial, with re-treatment at 4 weeks, 32% of the high dose and 40% of the low dose participants dropped out of the study [51]. Extrapolating from pharmacokinetic data derived from those with alcohol dependence, re-treatment of those with opioid dependence with injectable naltrexone would be required on a monthly basis [106, 210]. Therefore, although these newer sustained-release preparations help to overcome the issue of non-compliance for short periods (typically 21-28 days), they still incur significant levels of attrition with patients required to return every 3-4 weeks for re-treatment. Longer lasting sustained-release preparations might offer advantages for individual patients and the community.

An implantable naltrexone formula (GoMedical Industries Pty Ltd) has been used under the Commonwealth Therapeutic Goods Administration Special Access Scheme (“Compassionate Guidelines”) in Western Australia since August 2000. GoMedical implants in humans maintain naltrexone at therapeutic levels, i.e. above 2 ng/ml, for approximately 5.5 months, and 1 ng/ml for 9 months [147-149], and are associated with a significant reduction in hospital presentations and accidental overdose [159, 160]. However, there is concern regarding an increased risk of accidental overdose if relapse to opioids were to follow maintenance treatment with naltrexone [58, 265, 315, 336, 349, 370]. 

Fatal and non-fatal overdoses may be prevalent with naltrexone because, being an antagonist, it reduces tolerance to opioids; in addition, chronic exposure may result in up-regulation of opioid receptors thereby increasing sensitivity [72, 390]. Increased risk of mortality due to reduced tolerance has been shown in humans following periods of abstinence, such as following incarceration [58] but, although receptor up-regulation has been demonstrated in animal models [361, 403], it is still unclear if it occurs in humans [7, 55, 390]. Furthermore, naltrexone has been shown to suppress the *subjective* effects of opioids more than *objective* physiological effects [334, 376]. This dichotomy may increase the chance of an overdose as the user does not receive the expected level of feedback from a given level of opioid use, but still experiences physiological effects such as respiratory depression [72]. 

#### 2.3.2 Role of Naltrexone in Treatment of Dependence During Pregnancy

Naltrexone is not recommended for use during pregnancy due to lack of data on prenatal exposure and in Australia is classified as a B3 drug (Table **1**) – one where “…studies in animals have shown evidence of an increased occurrence of fetal damage, the significance of which is considered uncertain in humans” [38]. The FDA classifies naltrexone in the same category (C) as methadone and buprenorphine (Table **1**). However, because termination of maintenance therapies may be associated with relapse to heroin use, some pregnant women elect to remain on oral naltrexone during at least part, and some for the entirety, of their pregnancies [158]. One potential reason for remaining on naltrexone is that its lack of agonist activity circumvents neonatal abstinence syndrome [191, 427]. Detoxification of pregnant women onto oral or implanted naltrexone maintenance has also occurred in 6 cases, when these patients were unable to stabilise onto other maintenance treatments [146, 156-158]. The efficacy of naltrexone for treatment of the opioid-dependent pregnant woman has not sufficiently been assessed; the limited studies did not comment on toxicological screening for substance abuse.

#### 2.3.3 Pharmacokinetics of Naltrexone During Pregnancy

Whilst transfer of naltrexone into breast milk has been shown in sheep and humans [39, 40], and placental transfer demonstrated in rat [406, 407], naltrexone transfer across the human placenta has not been reported. Indeed, transfer of both naltrexone and 6,β-naltrexol may be limited by their extensive conjugation with glucuronic acid thereby reducing their permeability [71, 211, 326, 380, 382]. However, conjugated naltrexone and 6,β-naltrexol may be transferred by some as yet unknown transport mechanism. Nevertheless unconjugated transfer presumably does occur in humans, as occurs for many other drugs [292, 371, 387]. A single case study confirmed that maternal naltrexone levels can be sustained above a therapeutically effective threshold of 2 ng/ml for at least the first 214 days of pregnancy, reflecting similar levels measured in non-pregnant patients [147-149]. Placental transfer of naltrexone occurs in rat [406, 407], and despite interspecies difference in placental morphology, transport and metabolism is functionally similar [262, 358]. Indeed, opioidergic drugs, including morphine and naloxone, which are very similar to naltrexone [26, 44], rapidly cross the human placenta and enter the fetal circulation [47, 57, 206]. Furthermore, the physiochemical properties of naltrexone, namely its alkalinity (pKa: 8.13), high lipid solubility (octanol/water distribution: 13.08) [200], small molecular weight (341.41 Da) [182], and low plasma protein binding of 21-8% [68, 241] are factors which facilitate placental transfer [71, 140, 296, 354]. In contrast, 6,β-naltrexol, is hydrophilic [374] and its placental transfer is likely to be minimal compared to naltrexone. Transfer of naltrexone and 6,β-naltrexol in human lactate is low with an estimated total relative infant dose of 1.06% in a 24 hour period. Moreover, whilst 1.1 ng/ml 6,β-naltrexol was detected in neonatal plasma, no naltrexone was identified [40].

#### 2.3.4 Naltrexone and Neonatal Outcomes

Few data are available on the impact of oral or implantable naltrexone exposure and feto-maternal complications in pregnant humans [146, 149, 156-158]. Whilst all were co-authored by one of the contributors to this review (GKH), they include data from Australia, United Kingdom and Portugal and are the only publications on maternal and neonatal outcomes in humans following naltrexoneexposure during pregnancy. Australian cases were treated through the Australian Medical Procedures Research Foundation (AMPRF): until August 1999, oral naltrexone was only available in Western Australia (WA) from AMPRF. Clinic records indicate that 1196 patients had oral naltrexone maintenance between June 1997 and August 1999. A review of the first 300 patients indicated that 136 (45.3%) were females of reproductive age [150].

To January 2006, treatment files (AMPRF) indicated that 46 pregnant women treated with oral naltrexone had maintained contact with the clinic at time of birth (GKH, personal communication). Data on 10 cases treated with oral naltrexone in Australia, Portugal and United Kingdom indicate that outcomes of the seven births delivered at the time of study were generally unremarkable in terms of gestation age (between 38-40 weeks), good birth weight, head circumference, and APGAR scores. This was with the exception of one case in which the baby was induced at 36 weeks (resulting in low birth weight) due to maternal hypertension, and one other case of a baby weighing 2626 g at birth, born to a mother under 40 kg before conception and a father under 50 kg. Periodic foetal monitoring of the remaining three cases at the time showed normal development [158]. As the authors admit, the data may be biased since it only involved patients who maintained contact with the clinic, a factor which is known to improve outcome [221, 228]. No post-birth follow-up has taken place on any infants exposed during pregnancy. There is no indication as to the outcome in those who failed to maintain contact.

To January 2006 in Western Australia, approximately 650 women of reproductive age, had undergone treatment with the GoMedical naltrexone implant and, of these, 43 pregnant women were in contact with the drug treatment clinic at the time of birth (GKH, personal communication). Similar to oral naltrexone, review of the first nine neonatal cases with exposure at different stages of pregnancy for different patients indicate that neonatal and obstetric outcome was unremarkable. Some statistics of interest were: half (4) of the case babies were delivered *via* spontaneous vaginal delivery, gestation age ranging between 37.5 and 41 weeks, birth weight between 2720 g and 3845 g, length 47-53cm, head circumference 31-35.3 cm, and 5-minute APGAR scores of at least 9 [146, 156]. Again, these cases may be biased to more positive outcomes in patients who maintained contact with the drug treatment service. Taken together, the reports suggest no adverse effects on neonates from naltrexone exposure during pregnancy in humans. 

#### 2.3.5 Implantable Naltrexone Versus Methadone Maintenance

Obstetric and neonatal outcomes in a sequential cohort of seventeen of the above 43 heroin-dependent pregnant women managed by naltrexone implant have been compared with ninety women receiving MMT [157]. Following MMT,deliveries prior to 37 weeks (24%) and low birthweight (<2500 g, 23%) were both significantly greater (p<0.001) compared to Australian national data for the normal population (delivery prior to 37 weeks: 5.6%; birthweight<2500 g: 4.6%) [317]. By contrast, there was no significant difference between naltrexone implant-managed women (delivery prior to 37 weeks: 5.9%; birthweight<2500 g: 11.7%) when compared to National data [157]. In addition, the difficulty of adjusting to extra-uterine life for babies born to mothers receiving MMT was indicated by significantly worse one minute APGAR scores compared to infants of mothers treated with a naltrexone implant (p<0.005). However, the lack of significance with regards to adverse neonatal effects from naltrexone exposure during pregnancy could be a consequence of limited statistical power from the small sample size of 17.

## 3 ANIMAL STUDIES

To appropriately reflect the clinical situation of drug use during pregnancy, a number of factors need to be considered in animals studies. First, due to interspecies differences in pharmacokinetics, delivering a clinically comparable drug dosage in animal studies is not simply a case of using relative dose-to-weight ratio, but requires consideration of blood levels, duration of drug retention, and fluctuation from repeated dosing. In comparison to humans, where drug fluctuations tend to be minimal and blood levels are maintained within the therapeutic range [164], laboratory animals generally have a much higher rate of drug elimination [278], and produce a series of narrow and high-drug concentration peaks. Although peak plasma drug concentrations can be matched between animals and humans, the duration of clinically relevant blood levels is often harder to control [162, 164]. Second, the route of administration, and whether it is delivered maternally or directly to the offspring, will also influence the extent of offspring exposure. Therefore, the dose, route of administration, and the total exposure (reflected by the area under the concentration-time curve) should be assessed [168, 278]. Third, interspecies differences in parameters such as the onset, duration and intensity of tolerance, withdrawal, stress, and ontogenic response to a given dose of drug may also account for differences between human and animal offspring development [168, 408, 409]. 

Fourth, the period of exposure during development will influence outcome. Different species have different absolute developmental timetables. Nevertheless, mammals develop to a relative common timetable defined by the caecal period, namely the length of time from conception to eye-opening [77, 322]. In humans eye-opening occurs during the third trimester (182 days), but in rat occurs ~2 weeks after birth (gestation = 21 days), i.e. at post-conceptional day 36 days [59]. For example, the corpus callosum in rat appears at 18.5 days post-conception, and in humans appears at 87.5 days post-conception, respectively corresponding with 51% and 48% of the caecal period [59]. Likewise, the end of rapid axon loss in the nervous system of rats occurs at day 27 post-conception, and in humans at 136 days, which both correspond to 75% of the caecal period [77].

Fifth, when using animal models to study the direct, biological effects of a drug on development, it is desirable to control for potential indirect drug effects. For example, deprivation of nutrition has a profound effect on development and may result from a number of factors related to drug exposure, such as drug-induced appetite suppression, nutritional value of the milk and maternal behaviour [37, 99, 168, 408]. Prenatal drug manipulations of pregnant rats can modify maternal behaviour and so affect developmental parameters in offspring. These include circadian rhythms, milk consumption, activity level, heart rates, oxygen intake and growth hormone [168]. The effects can be controlled for prenatally by introducing a pair-fed control group, which involves matching the food consumption to that of the experimental group [162]; postnatally, drug-induced maternal effects can be isolated by cross-fostering [162], involving transfer of offspring from drug treated-mothers to non-treated mothers, and *vice-versa* [121]. Furthermore, in animals which typically birth multiple offspring, a drug may affect litter size, which would alter sibling competition with, for example, larger litter sizes decreasing nutritional distribution during gestation and lactation; a small litter size is also sub-optimal for offspring nutritional intake as reduced suckling limits maternal milk production and sustenance [173, 271, 287]. Such effects can be overcome by culling to an optimal litter size which in rat, for example, is 8-10 pups.

### 3.1 Methadone

#### 3.1.1 Doses, Metabolism and Transfer

In humans, daily oral methadone dosing, combined with the long terminal half-life (24 h), avoids withdrawal syndrome and results in continuous exposure of the neonate. However, in the pregnant rat, methadone metabolism is rapid (terminal half-life: 3-5 hours) and once-daily dosing can result in daily maternal and offspring withdrawal [163]. To achieve clinically relevant blood levels (150-500 ng/ml) [101, 135] in rats, a daily oral dose of approximately 12.5 mg/kg is required [166]. However, in most studies (Table **2**), a daily dose of 5 mg/kg of methadone was administered maternally, indicating an apparent “under-dosing”. However, compared to the clinical situation of oral dosing, intraperitoneal injections were used which result in high plasma levels, as first-pass hepatic metabolism is evaded. Furthermore, intraperitoneal injections can result in extraplacental transfer directly from the peritoneal cavity to fetal tissues and fluids [166, 300], which may account for increased exposure compared to other routes of administration. Nonetheless, in rat, methadone crosses the placenta, enters the fetal circulation [166] and transfers to the lactate [409]. However, in most studies, blood plasma levels were not reported and interspecies differences in the rate of metabolism of methadone means that it is hard to replicate fetal plasma exposure found in humans [105, 107, 233]. Regardless, ontogenesis in rat is affected by a sub-therapeutically relevant dose (5 mg/kg) of methadone (Table **2**).

#### 3.1.2 Effects on Offspring: Growth and Brain Development

Decreased birthweights found in humans are consistent with animal studies which report that gestational methadone exposure reduces growth [99, 166, 256, 410]. An early study on maternally administered methadone (10-13 mg/kg/day) in primates showed significantly reduced birthweights in the offspring [135]. In rat, a number of deleterious effects are associated with perinatal methadone exposure including increased mortality of mothers [167] and offspring [34, 166, 408], as well as low birthweights and growth retardation [41, 166, 257, 408]. Prenatal methadone also delays the initial onset of various physical characteristics in rats, such as the appearance of hair and opening of the vagina, eyes and ears [103, 253, 383, 412].

The inhibitory growth effects are, to a certain extent dose-dependent [41, 166, 420], and influence the animals as a whole as well as individual organs, with some being affected more than others [257]. In particular, the central nervous system (CNS) is a sensitive target [409, 428]. Adverse effects include reduced brain size and weight, decreases in cell density and total cell number, as well as neurochemical alterations (Table **2**). In particular, there are consistent decreases in protein, DNA, RNA and neurotransmitter content (serotonin, dopamine, norepinephrine) [87, 145, 253, 301, 344], enzymatic protein levels (tyrosine hydroxylase, acetylcholine-esterase, choline acetyltransferase, ornithine decarboxylase) [87, 224, 327, 338, 342, 343], and receptor expression (β-adrenergic) [66]. Moreover, some of these effects are region-specific and age-dependent; for example, norepinephrine content has been found to decrease in the forebrain but not the hindbrain [253], and whilst acetylcholine-esterase levels following prenatal methadone exposure are normal at birth, they are reduced at 21-days postnatal [87]. Acetylcholine, enkephalin and nerve growth factor are also reduced at birth following exposure during pregnancy [123, 319, 366, 399].

#### 3.1.3 Effects on Offspring: Behaviour

Methadone during prenatal development affects postnatal behaviour. The withdrawal syndrome has been observed in newborn rat offspring following maternal treatment with an osmotic minipump throughout pregnancy; NAS was precipitated by a challenge of the opioid antagonist, naltrexone and observed as increased motor activity and vocalisation [13]. Neonatal abstinence syndrome was also associated with alterations in the latency of visually evoked potentials [305]. Young and mature adult offspring also displayed increased withdrawal-like symptoms in comparison to control animals, such as head and body shakes [416].

Prenatal exposure to methadone also delayed development of reflex and motor coordination [253, 383, 412]. In most studies, there was an initial decrease in open field ambulation and exploration in rat offspring up to 45 days postnatal [103, 122, 253, 261, 383, 436]. Conversely, there was a consistent increase in open-field activity in rat offspring after 45 days postnatal [122, 347, 436]. 

Methadone exposure during pregnancyhas also been found to affect analgesic responses later in life, although the effect has been variable; some studies report an increase [414, 415, 417, 426] and another a decrease [143, 416]. Moreover, the analgesic effects of drugs, such as amphetamine and methadone, were greater in methadone-exposed offspring [415, 426]. There was also an increased propensity to self-administer oral morphine [144, 261]. Furthermore, decreased latency to step down from a platform could indicate increased anxiety [436]. Consistent with human studies, memory and learning tests revealed poorer cognitive skills [372, 413, 437]. Another study demonstrated that decreased discrimination learning was dependent on the period of methadone exposure; rats exposed prenatally or postnatally displayed an effect, those exposed both pre- and post-natally showed none [372].

### 3.2 Buprenorphine 

Animal studies of the ontogenic effects of buprenorphine are relatively limited in comparison to more extensive studies on the effects of methadone or naltrexone. The pharmacokinetics of buprenorphine in pregnant rats is yet to be investigated.

#### 3.2.1 Effects on Offspring: Growth and Brain Development

Maternal exposure to buprenorphine (0.67-2 mg/kg) during pregnancy results in an increase in offspring mortality within 6 days of birth [83, 320, 366], whilst resorptions were unaffected [165]. In contrast to methadone, buprenorphine increases the duration of gestation [83]. It is unclear what impact prenatal buprenorphine (0.07 or 0.67 mg/kg/day) has on birthweight with one study reporting no effect [165], and another a decrease [83]. However, prenatal exposure to buprenorphine results in decreased postnatal weight gain [320].

There are more data on biochemical and protein changes following prenatal exposure to buprenorphine (Table **3**). μ-opioid receptor expression is down-regulated in newborn rat offspring [19], but this effect is transient and diminishes by postnatal day 7 [20]. Whilst these effects were associated with low doses of buprenorphine (0.15-0.5 mg/kg/day), a higher dose (2.5 mg/kg/day) resulted in κ-opioid receptors up-regulation in neonatal offspring [19]. In both studies, the ligand binding affinities (Kd) for μ- and κ-opioid receptors were unaffected. However, in 2-day-old offspring exposed to buprenorphine prenatally, receptor function is diminished, as evidenced by decreased coupling activity of the stimulatory G-protein following agonist stimulation of μ- (ventral tegmental area) and N/OFQ-opioid receptors (lateral septum and nucleus accumbens), [141]. The decline in receptor activity was transient, diminishing by postnatal day 7, and a gender related difference was reported, with males having greater decreases in receptor function. 

Prenatal buprenorphine exposure also results in a decrease in choline acetyltransferase mRNA in postnatal offspring [318] although striatal mRNA levels for the μ-, δ- and κ-opioid receptors are unaffected [19, 366]. Administration of 0.3 mg/kg of buprenorphine during gestation resulted in a decrease of striatal acetylcholine content in 4 day old rat offspring, although there was an overshoot with greater levels than controls at 21 days [319]. However, treatment with a higher dose (3 mg/kg) decreased striatal acetylcholine content in both 4- and 21-day-old rat offspring. Prenatal buprenorphine does not affect NGF mRNA levels in postnatal day 10 [318] although striatal NGF content was decreased [318, 321]. 

The effects of postnatal exposure have also been investigated, but the studies are few and did not show significant effects [165, 169]. Due to the mixed agonist-antagonist action of buprenorphine, translation of its mechanism of action has been limited because of the following: 1) the pharmacokinetics of buprenorphine in the pregnant rat, or any other animal, does not appear to have been evaluated; 2) the ontogenic role of the κ-opioid receptor requires clarification, as does the pharmacological action of buprenorphine at this receptor; and 3) levels change during each 24 hour cycle of a daily dosing regime; initial high doses result in antagonism at the μ-opioid receptor and later low doses result in agonist action. Presumably, during each 24 hour period, the developmental effects would alternate as blood levels fluctuate. Avoidance of fluctuating levels using constant infusion may help to separate the agonist/antagonist effects of buprenorphine. However, such studies would be difficult during lacation because maternal delivery *via* the milk would also fluctuate; furthermore, constant infusion in neonatal rats is difficult. Differential impact on receptor expression has been demonstrated with up-regulation of κ- and δ-opioid receptors and down-regulation of µ-opioid receptors from daily intraperitoneal injections ≥0.5 mg/kg during the postnatal period [18]. Gestational exposure was shown to have similar effect on κ- and µ-opioid receptors, when evaluated at the time of birth [19]. These effects are similar to that expected from methadone at μ-opioid receptors, although with less efficacy, and similar to naltrexone at the kappa-opioid receptor. It is unknown what effect buprenorphine has on the ζ-opioid receptor; considering the significance of this receptor’s role in ontogenesis, this warrants further investigation. 

#### 3.2.2 Effects on Offspring: Behaviour

Following buprenorphine exposure, a number of behavioural changes are detected including decreased sensitivity to morphine, increased morphine-withdrawal and an earlier onset of the righting reflex, as well as a delayed ear opening [320]. A gender-related difference in preference for saccharine is diminished following offspring exposure to prenatal buprenorphine [14], although rest-activity was unaffected [165]. The effects could be explained by parental behaviour (i.e. pup retrieval, grooming and hovering/crouching over the pups), being impaired during the prenatal or postnatal period [14].

### 3.3 Naltrexone 

While the initial clinical data may appear to provide some evidence that outcomes associated with both implant and oral naltrexone may be as good, if not better, than observed with methadone, research data from animal work raises concern.

#### 3.3.1 Doses, Metabolism and Transfer

In rat, naltrexone is metabolised to 6,β-naltrexol and the minor metabolites *via* the same metabolic pathways [380]. The elimination half-life is 4.6 hours [402] being very similar to human (4 h). However, relative to naltrexone, small amounts of 6,β-naltrexol are found in rat plasma and urine, whereas significant quantities are found in humans [240]. Notwithstanding the difference, naltrexone-plasma levels of 2 ng/ml in rat have been shown to block the analgesic effects of morphine (plasma concentration: >1000 ng/ml) [402], which parallels the pharmacodynamic efficacy of naltrexone in humans [15, 181]. 

Most experiments used high doses of naltrexone (50 mg/kg/day) that are above those used clinically and may, therefore, be misleading. Naltrexone levels which affect development are reportedly small (0.05-2.5% of the LD_50_ in adult rats) [428]. With an LD_50_ of approximately 2000 mg/kg in adult rats [31, 439], the dosages required to induce and effect in rats are 1-50 mg/kg, with 1 mg/kg considered a low dose (Table **5**), and 50 mg/kg a high dose (Table **4**) [425]. 

Naltrexone transfer across the placenta has been established in rats [406, 407]. Naltrexone has not yet been measured in rat lactate. However, since naltrexone transfer to milk has been confirmed in humans and sheep, it is presumed that transfer into rat milk will also occur. Indeed, naltrexone has been detected in the blood of 4-week-old offspring when naltrexone was only delivered maternally^1^, suggesting that it is indeed transferred to the rat lactate and can therefore affect development after birth [39, 40].

#### 3.3.2 Effects on Offspring: Growth and Brain Development

The effects of naltrexone on growth are somewhat opposite to the suppression following prenatal exposure to methadone and other opioids [161, 223, 345, 421]. A number of studies indicate increased body weight and length and also increased dry and wet weights of body organs including the brain [254, 255, 442]. However, an increased level of complexity is that the effects of naltrexone on growth can be either stimulatory or inhibitory depending on the dose and its duration [423, 425, 428, 430], a scenario that is likely in the clinical setting. However, in these studies, blood plasma concentrations were not measured and have to be inferred from the dose regime. For example, in rat, daily administration of a high dose (50 mg/kg) of naltrexone, ensures continuous blockade of receptors and results in accelerated growth (Table **4**). In contrast, daily administration of a low dose (1 mg/kg) blocks receptors temporarily because the agent is eliminated before dosing the next day; this intermittent blockade has been associated with inhibition of growth (Table **5**). The inhibitory effect is similar to that seen following developmental exposure to an opioid agonist, such as morphine or methadone [431]. However, some studies adhering to similar dosing regimens report no effect [64, 137, 302], a discrepancy which has yet to be clarified.

Nevertheless, the duration of opioid receptor blockade appears to be more important in modulating development rather than its actual dose [425]. Multiple doses of naltrexone (3 mg/kg three times per day), which ensure continuous receptor blockade, accelerates body and brain development. By contrast, a single cumulatively equivalent dose (9 mg/kg once per day), which induces intermittent receptor blockade, has an inhibitory effect [425]. 

Intermittent blockade by antagonists can exert its effects *via* a mechanism termed *supersensitivity*, which is characterised by accentuation in receptor expression and function [17, 335, 428]. In the absence of activation, alterations in receptor structure can result in a change to a high agonist affinity state [309, 445]. In the high affinity state, a dose which previously elicited a response of a particular magnitude may induce a response of a much greater magnitude due to the higher incidence of binding and subsequent receptor activation. An increase in the number of opioid receptor binding sites also contributes to a higher incidence of binding and hence, supersensitivity to opioids [423, 428, 431]. Supersensitivity can also be associated with increased endogenous agonist levels and/or increased affinity, and/or efficacy of the receptor-agonist complex. Indeed, opioid antagonists are also known to increase the levels of endogenous opioids, such as β-endorphin [429]. Thus, compared to normal, in a supersensitive state, basal or elevated endogenous opioid levels can exert a greater inhibitory action, manifested by a reduced rate of cell proliferation and growth [428].

Experimental studies have yet to investigate the developmental effects of naltrexone delivered as a sustained-release implant or depot. GoMedical implants release approximately 220 μg/kg/day in humans and achieve plasma levels of 2-10 ng/ml [147-149]. The amount released is 4-5 times lower than the minimum dose that is thought to affect development and it is not yet known whether naltrexone delivered in this way will have an effect, an issue that warrants investigation. Nevertheless, one study showed that continuous naltrexone exposure (10 mg/kg/day *via* osmotic mini-pump) during the pre- and post-natal period resulted in increased thickness of the cerebral cortex and decreased, packing density although cortical cell number remained the same [337]. In contrast to accelerated growth from continuous prenatal exposure, it is hypothesised that, during the postnatal period, a maternally administered sustained-release naltrexone implant will do the opposite compared to intrauterine exposure, i.e. offspring will be exposed intermittently during feeding from the mother. Furthermore, as tolerance is not observed with naltrexone exposure [204, 266, 376], it is hypothesised that the resulting developmental effects would be influenced by the developmental phase taking place during exposure with differential opioid receptor expression playing a crucial role [11, 132].

Whilst developmental studies on naltrexone’s effect on neurochemistry are sparse, daily injections (50 mg/kg) of rat pups had variable effects on NGF, with decreases in cerebellum and increases in the hippocampus [268]. Exposure to 1 mg/kg also decreased NGF content in hippocampus, septum and neostriatum whereas 50 mg/kg had no effect on NGF content but decreased the number of NGF high affinity binding sites [298]. A lower dose (1 mg/kg) administered to pups from birth until day 21 decreased striatal and hippocampal serotonin levels in adulthood [379], following an increase in striatal serotonin at 22 days postnatal [63].

#### 3.3.3 Effects on Offspring: Behaviour

Behavioural differences are manifested at the cellular level by alterations in excitability and changes in the morphology and connectivity [348]. Daily injections into pregnant rats result in changed behaviour of offspring after birth, for example, decreased grooming, ambulation and defecation [254, 255, 442]. The effects of gestational and/or postnatal exposure to naltrexone on behaviour have been explored [8, 48, 65, 131, 202, 255, 258, 297, 427, 438]. Daily exposure to 50 mg/kg during the pre-weaning period accelerates the development of spontaneous motor and sensorimotor behaviours, namely initial appearance and the age at which 100% of the animals demonstrate a particular behaviour [255]. In contrast, daily exposure to 1 mg/kg delayed the initiation of developmental events such as ear opening, eye opening and walking. Whilst control and 1 mg/kg naltrexone groups are comparable in motor activity at 21 days, an increase of 32% in activity was recorded in animals of the 50 mg/kg group [427]. Mice administered with 10 mg/kg twice daily through-out gestation and lactation reared offspring who displayed increased locomotor activity in adulthood [258].

Naltrexone exposure during gestation and the post-weaning period decreased the sensitivity of the offspring to the analgesic effects of morphine [131, 297, 438]. Only one study has examined the behavioural effects of sustained-release naltrexone (*via* a minipump; 10 mg/kg/day), starting on day 17 of gestation [202]. As prenatal stress was included as a treatment in this paradigm, only a few independent effects of naltrexone on the offspring were identified. These included increased anxiety, decreased preference for saccharin and decreased sensitivity to pain. Increased anxiety and emotionality was found in another study exploring conditioned emotional response, although exposure to naltrexone was *via* daily maternal administration of 1 mg/kg throughout gestation [131]. Other behavioural effects of naltrexone exposure during development include facilitation of male sex behaviour as indicated by a shorter mount time [48], and reduced food intake after 14 days of postnatal exposure [8]. Another study in sheep showed that acute naltrexone administration (1.5 or 3 mg/kg, intrapeitoneal) within 24 hours of birth prevented lambs from recognising their mother, suggesting an interruption of bonding *via* the opioid reward system [340].

## CONCLUSIONS

Studies in humans to date have shown that MMT during pregnancy is associated with a number of adverse neonatal effects, including reduced birth-weight and the incidence of NAS. However, the negative outcomes associated with MMT outweigh the detrimental effects associated with heroin use during pregnancy, although whether these benefits are due to MMT or to improved psychosocial stability needs clarification through well-controlled randomised trials. There are an abundance of data from animal pregnancy studies that reflect many of the adverse effects seen in humans, many of which have involved clinically applicable dosing regimens. However, various aspects of MMT, such as the potential benefits of split-dosing and breast feeding to treat NAS have received little attention in animal research and warrant elucidation. 

Buprenorphine has arisen more recently as an alternative treatment for the pregnant addict and there is evidence to suggest a number of advantages over MMT, such as a reduced incidence and severity of NAS. However, both human and animal data are limited. Moreover, notwithstanding the complexity of interpreting the developmental effects of a partial agonist, both human and animal studies have highlighted detrimental neonatal outcomes. In particular, the differential pharmacological activity, which is dependent upon dose, needs to be investigated in terms of ontogeny, to determine which regimen offers optimal outcomes for the newborn.

An even more novel approach for treating the pregnant heroin user, using naltrexone, offers the advantage of evading withdrawal in the newborn and is accounted for by its lack of agonist activity. The advent of administering naltrexone as a sustained-release implantable preparation has resulted in an increase in the frequency of inadvertent exposure of the neonate to naltrexone. Nonetheless, based on human data, this mode of delivery is promising as early indications suggest an absence of detrimental neonatal outcomes, although the work urgently requires validation. Moreover, animal research, examining oral or injected naltrexone, has demonstrated that the developing neonate is affected following gestational exposure. The sustained-release implant described for use in humans results in chronic and low-dose exposure, a regimen which has yet to be applied in animal studies. Since animal studies using other dosing regimens raise concern, research on the sustained-release preparation is also necessary.

Pregnancy remains a unique situation where very important and considerable ethical judgment is required when commenting on pharmacological treatment and more animal data should be made available to determine which pharmacotherapy provides the optimal neonatal outcomes. Given the detrimental impact of illicit drug use during pregnancy, investigation of pharmacological treatment during pregnancy is very important, urgently needed and appropriate further investigation should be undertaken.

## Figures and Tables

**Table 1:** Overview of FDA (United States) and TGA (Australia) Pregnancy Classification Relevant to Methadone, Buprenorphine and Naltrexone

	Category Definition	Drug(s) in Category
FDA (United States)	C: “….either studies in animals have revealed adverse effects on the fetus (teratogenic or embryocidal effects or other) and there are no controlled studies in women, or studies in women and animals are not available.”	Methadone, Buprenorphine, Naltrexone
TGA (Australia)	B3: “Drugs which have been taken by only a limited number of pregnant women and women of childbearing age without an increase in the frequency of malformation or other direct or indirect harmful effects on the human fetus having been observed. Studies in animals have shown evidence of an increased occurrence of fetal damage, the significance of which is considered uncertain in humans.”	Naltrexone
	C: “Drugs which, owing to their pharmacological effects, have caused or may be suspected of causing harmful effects on the human fetus or neonate without causing malformations. These effects may be reversible. Accompanying texts should be consulted for further details.”	Buprenorphine, Methadone

**Table 2:** Summary of the Effects of Maternally Administered Methadone on the Development of the Rat Brain

Reference	Daily Dose (mg/kg)	Route of Admin.	Duration of Drug Exposure	Age of Offspring when Evaluated (days)	Developmental Effects (Compared to Controls)
[394]	5	s.c.	Gest	1 or 3	Reduced explant outgrowth size.
[408]	5	i.p.	Gest-lact or gest-lact + post-weaning (60 days)	0, 3, 6, 10, 15, 21 or 60	Decreased brain & cerebellar wet weights & reducedcerebral & cerebellar widths.
[409]	5	i.p.	Gest, lact or gest-lact	0, 3, 6, 10, 15, 21 or 60	Decreased cerebellar wet weight & brain length.
[410]	5	i.p.	Gest, lact or gest-lact	21	Decreased brain wet weights & DNA content.
[257]	5	i.p.	Gest, lact or gest-lact	21	Reduced brain wet & dry weights.Smaller head diameters.
[411]	5	i.p.	Gest, lact or gest-lact	10, 21 or 60	Decreased brain & cerebellar DNA content.
[99]	1-5 (twice daily)	s.c.	Gest day 13 to birth	7, 14, 21 or 28	Reduction in brain wet weight, cortical thickness &the number of cells in the neocortex.
[256]	5	i.p.	Gest, lact or gest-lact	60	Decreased head diameter & brain DNA content.
[418]	5	i.p.	Gest, lact or gest-lact	21 or 60	Decreased brain & cerebellar wet weights.Reduced DNA, RNA & protein concentration in brain & cerebellum
[420]	5	i.p.	Gest or lact	21	Reduced area in the pyramis (cerebellar lobule) & decreases in the total number & density of internal granule neurons.
[424]	5	i.p.	Gest-lact	21	Decreased brain wet weight & length.
[318]	9	s.c. OMP	Gest	4,10 or 22	ACh & NGF content reduced in striatal neurons,which are smaller in size.

Gest = gestation, lact = lactation, s.c. = subcutaneous, i.p. = intraperitoneal, OMP = osmotic minipump, ACh = acetylcholine and NGF = nerve growth factor.

**Table 3:** Summary of the Effects of Maternally Administered Buprenorphine on the Development of the Rat Brain

Reference	Daily Dose(mg/kg)	Route of Admin.	Duration of Drug Exposure	Age of Offspring when Evaluated (days)	Developmental Effects(Compared to Controls)
[336]	1 or 2	s.c.	Gest	21	No effecton met- or leu-enkephalin levels.
[17]	0.5	i.p.	E14-22	1	Decreasedµ-receptor Bmax in brain membranes.
				7	No effecton µ-receptor Bmax in brain membranes.
				1 & 7	No effect on µ-receptor Kd.
[165]	0.3, 1 or 3	s.c. OMP	E8-E22	0, 22 or 30	No effect on rest-activity cycle.
[14]	0.15 or 0.3	s.c.	E6-20	1, 28, 50	Impaired parental behaviour (0.6), expected sex difference for saccharine preference diminished (males consuming more in 0.6 grp).
[19]	2.5	s.c.	E6-20	1	Up-regulation of κ-receptors.
	0.15, 0.3 or 2.5				Down-regulation of µ-receptor. No effect on µ- nor κ-receptor Kd & mRNA.
[321]	1.5	s.c. OMP	E7-22, P0-10 & E7-P10	10	Decreased striatal NGF content by >40%.
[318]	-	-	Gest	10 & 22	Decreased striatal choline acetyltransferase mRNA.
[320]	0.3, 1 or 3	s.c. OMP	E7-P13, P0-13 or E7-P13	0-21	Increase in morphine ED25 pre &/or post (larger dose=large decrease in sensitivity). Increased withdrawal score (0.3 & 3). Altered timing of behavioural characteristics.
[399]	1.5	s.c. OMP	E7-P0, P0-10 or E7-P10	10	Decreased striatal NGF.
				4 or 10	No effect on NGF mRNA.
[319]	0.3	s.c. OMP	E7-P0	4	Decreasedstriatal ACh.
			E7-P0	21	Increasedstriatal ACh (males).
			P0-21		
	3		P0-21	4	Decreasedstriatal ACh.
			E7-P21	21	
[141]	0.5 or 1	s.c. OMP	E10-P7	2	Decreasedµ-receptor GTPγS binding in ventral tegmental area.
				7	No effect on µ- nor N/OFQ-receptor GTPγS binding.
	1			2	Increased N/OFQ-receptor GTPγS binding in nucleus accumbens & lateral septum (males).

Gest = gestation, E = embryonic day, P = postnatal day, s.c. = subcutaneous, i.p. = intraperitoneal, OMP = osmotic minipump, ACh = acetylcholine, NGF = nerve growth factor and GTPγS = guanosine triphosphate gamma stimulatory protein.

**Table 4:** Summary of the Effects of High/Continuous Doses of Naltrexone on Postnatal Development of the Rat Brain when Administered Directly (with One Exception of Maternal Administration)

Reference	Daily Dose (mg/kg)	Route of Admin.	Duration of Drug Exposure	Age of Offspring when Evaluated (days)	Developmental Effects (Compared to Controls)
[422]	50	s.c.	Birth until 21 days postnatal	21	Greater brain & cerebellar wet weights. Thickersomatosensory cortex. Cerebellum total area larger.More glial cells & granule neurons.
[423]	50	s.c.	Birth until 21 days postnatal	21	Increased brain wet weight.
[425]	3 (3 times daily), 20, 50 or 100	s.c.	Birth until 21 days postnatal	21	Increased brain wet weight.
[428]	50	s.c.	Birth until 21 days postnatal	21	Increased brain wet & dry weights.
[429]	50	s.c.	Birth until 21 days postnatal	21	Increase in number of granule cells of dentate gyrus. Increase in cerebral & cortical width & area.
[430]	50	s.c.	Birth until 21 days postnatal	21	Increases in cerebellar surface dimensions, neural cell numbers, density & size.
[431]	50	s.c.	Once on postnatal day 6	6	Increased proliferation (labeling index) in cerebellum.
[133] & [134]	50	s.c.	Birth until 10 days postnatal	10	Increased purkinje cell dendritic length & spine concentration of granule cells.
			Birth until 21 days postnatal	21	Increased cell dendritic length & spine concentration in hippocampus.
[174]	50	i.p.	Once on postnatal day 1	1	Increased proliferation (labelling index) in ganglion cell layer of the retina
[254]	50	Maternal i.p.	Daily throughout gestation	0, 10 & 21	Increased brain dry & wet weights.

Admin. = administration, s.c. = subcutaneous, and i.p. = intraperitoneal.

**Table 5:** Summary of the Effects of Low/Intermittent Doses of Naltrexone on Postnatal Development of the Rat Brain when Administered Directly

Reference	Daily Dose (mg/kg)	Route of Admin.	Duration of Drug Exposure	Age of Offspring when Evaluated (days)	Developmental Effects (Compared to Controls)
[423]	1	s.c.	Birth until 21 days postnatal	21	Decreased brain wet weight.
[425]	0.1, 1 or 9	s.c.	Birth until 21 days postnatal	21	Decreased brain wet weight.
[428]	1	s.c.	Birth until 21 days postnatal	21	Decreased brain wet & dry weights.
[429]	1	s.c.	Birth until 21 days postnatal	21	Increase in cell density, with decreased macroscopic area of hippocampus. Decreased cerebral area & width.
[430]	1	s.c.	Birth until 21 days postnatal	21	Decreases in cerebellar surface dimensions & density of internal granule cells.
[431]	1	s.c.	Once on postnatal day 6	6	Decreased proliferation (labeling index) in cerebellum.
[134]	1	s.c.	Birth until 10 or 21 days postnatal	10 or 21	Subnormal dendrite length & spine growth, predominant by day 21.

Admin. = administration, s.c. = subcutaneous, and i.p. = intraperitoneal.

## Footnotes

1 Farid, W.O., Tait, R.J., Hulse, G.K., Dunlop, S.A. (2006) The effects of maternally administered methadone and naltrexone on the developing brain (Poster Abstract).*Proceedings of the Australian Neuroscience Society: 26th Annual Meeting,***17,** 76.

## ABBREVIATIONS

AMPRF: = Australian Medical Procedures Research Foundation
BBB: = Blood brain barrier
CYP: = cytochrome P_450_
EDDP: = 2-ethylidene-1,5-dimethyl-3,3-diphenylpyrrolidine
EMDP: = 2-ethyl-5-methy-3,3-diphenyl-pyrroline
FDA: = Food and Drug Administration
MMT: = methadone maintenance treatment
NAS: = neonatal abstinence syndrome
OGFr: = opioid growth factor receptor
TGA: = Therapeutic Goods Administration
VEP: = visual evoked potential
WA: = Western Australia
